# The Ontogeny and Brain Distribution Dynamics of the Appetite Regulators NPY, CART and pOX in Larval Atlantic Cod (*Gadus morhua* L.)

**DOI:** 10.1371/journal.pone.0153743

**Published:** 2016-04-21

**Authors:** Hoang T. M. D. Le, Anna Rita Angotzi, Lars O. E. Ebbesson, Ørjan Karlsen, Ivar Rønnestad

**Affiliations:** 1 Department of Biology, University of Bergen, Bergen, Norway; 2 Uni Research Environment, Uni Research AS, Bergen, Norway; 3 Institute of Marine Research, Storebø, Norway; Nord University, NORWAY

## Abstract

Similar to many marine teleost species, Atlantic cod undergo remarkable physiological changes during the early life stages with concurrent and profound changes in feeding biology and ecology. In contrast to the digestive system, very little is known about the ontogeny and the localization of the centers that control appetite and feed ingestion in the developing brain of fish. We examined the expression patterns of three appetite regulating factors (orexigenic: neuropeptide Y, NPY; prepro-orexin, pOX and anorexigenic: cocaine- and amphetamine-regulated transcript, CART) in discrete brain regions of developing Atlantic cod using chromogenic and double fluorescent *in situ* hybridization. Differential temporal and spatial expression patterns for each appetite regulator were found from first feeding (4 days post hatch; dph) to juvenile stage (76 dph). Neurons expressing NPY mRNA were detected in the telencephalon (highest expression), diencephalon, and optic tectum from 4 dph onward. CART mRNA expression had a wider distribution along the anterior-posterior brain axis, including both telencephalon and diencephalon from 4 dph. From 46 dph, CART transcripts were also detected in the olfactory bulb, region of the nucleus of medial longitudinal fascicle, optic tectum and midbrain tegmentum. At 4 and 20 dph, pOX mRNA expression was exclusively found in the preoptic region, but extended to the hypothalamus at 46 and 76 dph. Co-expression of both CART and pOX genes were also observed in several hypothalamic neurons throughout larval development. Our results show that both orexigenic and anorexigenic factors are present in the telencephalon, diencephalon and mesencephalon in cod larvae. The telencephalon mostly contains key factors of hunger control (NPY), while the diencephalon, and particularly the hypothalamus may have a more complex role in modulating the multifunctional control of appetite in this species. As the larvae develop, the overall progression in temporal and spatial complexity of NPY, CART and pOX mRNAs expression might be correlated to the maturation of appetite control regulation. These observations suggest that teleost larvae continue to develop the regulatory networks underlying appetite control after onset of exogenous feeding.

## Introduction

Atlantic cod (*Gadus morhua*) has a key role in North Atlantic fisheries and several major attempts to introduce cod into aquaculture have occurred over the last decades [1, 2]. Following the recent sequencing of the Atlantic cod genome [3], this species is also being explored as a model for understanding the genetic control of development and physiology in relation to environmental and nutritional factors [4]. During the early life stages, Atlantic cod undergo significant changes in feeding biology. At the onset of first feeding (4 dph), the larvae are 4–5 mm long and possess a simple and straight gut lumen. The intestine develops both in length and complexity including the development of elaborate folding of the mucosa layer. The stomach starts to differentiate from the foregut at about 25 dph [5, 6] although it takes a long time (>50 dph) before it becomes fully functional [7]. Larvae have been observed to continue to feed despite a full gut, especially just after onset of exogenous feeding [8]. These observations raise the question whether appetite control (particularly satiety) and digestive processes are fully functional in early larval stages [8]. The neuronal network underlying appetite control in vertebrates includes both orexigenic and anorexigenic signals, mainly neuropeptides produced in the brain, where the hypothalamus plays a pivotal role in the regulation of feeding [9–11]. Neuropeptide Y (NPY) appears to stimulate feeding behavior in several studies on teleost fish [11]. In Atlantic cod, RT-PCR analysis on whole larvae demonstrated NPY mRNA expression as early as 3 dph and a decrease during further development with the final assessment in larvae aged 60 dph [12]. Cocaine- and amphetamine-regulated transcript (CART) is a well-known anorexigenic factor that mediates feeding behavior and body-weight control in both mammals [13] and fish [11]. In Atlantic cod, the first detection of CART mRNA expression was found at 3 dph [12], i.e. at onset of first exogenous feeding. The Orexin A (hypocretin-1) and Orexin B (hypocretin-2) are two orexigenic neuropeptides produced from a single gene precursor known as prepro-orexin (pOX) [14]. In the teleost fish investigated so far, the onset of pOX expression occurs at very early embryonic stages [15]. Consistently, RT-PCR studies in Atlantic cod revealed pOX expression as early as cleavage stage [16].

Although recent studies have described the distribution of the neuropeptides NPY, CART, and pOX in the brain of adult Atlantic cod and other teleost fish [17–21], the spatial and temporal mRNA distribution of these genes during the critical first feeding stages and subsequent development are unknown. In the present study, we describe the mRNA-expression patterns of NPY, CART and pOX in the brain of Atlantic cod from first feeding (4dph) to 76 dph, with emphasis on homologous regions to mammalian appetite control regions located in both forebrain and midbrain. We further investigated coexpression of orexigenic and anorexigenic factors (NPY/CART, and pOX/CART) in these brain regions.

## Materials and Methods

### Fish material and sampling

The study was performed at Austevoll Research station (outside Bergen, Norway). The larvae originated from five-year old Atlantic cod broodstock (3–5 kg), grand-offspring of wild fish raised in net-pens at ambient temperature and salinity in Parisvannet, Norway (60°37’N, 4°48’E). The broodstocks were kept at 1:1 sex ratio and fed *ad libitum* on artificial feed three times a week. Eggs stripped from females were fertilized and incubated at 6°C under a 12L:12D light regime. The larvae were transferred to start feeding units and maintained according to best practice protocols at Institute of Marine Research (IMR) [22] at 8°C with a 16L:8D photoperiod and fed three meals of zooplankton per day [22]. The zooplanktons were filtered from a nearby pond, and from 4 to 34 days post hatching (dph) consisted of nauplii and copepodites (5–34 ind./mL), and from 35 to 46 dph mainly copepodities (3–7 ind./mL). From 47 to 55 dph, the larvae were fed with copepodities (3–7 ind./mL) and started weaning by AgloNorse^®^ 300–500 μm (Tromsø Fiskeindustri AS, Tromsø, Norway). From 56 dph, larvae were fed on only AgloNorse^®^ 400–600 μm. Experiments were carried out in triplicate tanks.

The mean standard length of the larvae sampled at 4, 20, 46 and 76 dph were 4.5, 5.9, 14.1 and 37.6 mm, indicating that they were in the Stages 1, 2, 4 and juvenile according to Sæle et al. (Ø. Sæle, NIFES, Bergen, Norway, personal communication). Cod larvae in Stage 1 are characterized an open mouth, pigmented eyes and the utilization about ¾ of the yolk sac has been used. Stage 2 is prior to metamorphosis, the ossification in the head is dominated by the jaws, and some ossification is also observed in the hyoid arch, the gill arches and some parts of the neurocranium. In Stage 4, after metamorphosis, the fins are developed but unpigmented, flexion is present, and most of the bones in the head are being ossified. The stomach is differentiated and the larval fin fold has disappeared. In the juvenile fish the pigmentation is complete.

### Identification and cloning of NPY, CART and pOX genes

NPY, CART, and pOX sequences were retrieved from GeneBank sequence database (NCBI) with accession number AY822596, DQ167209, and DQ486137, respectively. cDNA sequence fragments of NPY (346 bp), CART (300 bp), and pOX (453bp) were synthesized from total RNA extracted from cod brain, using oligo(dT)12-18 primer and Superscript III (Invitrogen, Carlsbad, CA, USA), by standard procedures. The primers used were: ggaactctgaccgagggat NPY(F) and gccctctgatgacaaatca NPY(R) for NPY; gagtgtggaccagagccttg CART(F) and gcagtcacacatcttcccaat CART(R) for CART; aatgaagtggtcctccacagtgt Orex(F) and tcaagcggtgaagtcttgctgc Orex(R) for pOX. PCR amplification was performed with an initial step of 94°C for 5 min, 30 cycles of 94°C for 25s, 55°C for 30s, 68°C for 90s and extension at 68°C for 7 min. PCR products were purified using QIAquick Gel Extraction Kit (Qiagen, Hilden, Germany) and cloned into TOPO cloning vector (Invitrogen). The inserts were sequenced at the University of Bergen Sequencing Facility, and used to synthesize antisense and sense (control) probes with digoxigenin (DIG RNA labeling mix, Roche Diagnostic) and fluorescein (Fluorescein RNA Labeling Mix, Roche Diagnostic) haptens for ISH methods.

### Tissue preparation for cryosectioning

Cod larvae (4, 20, 46, 76 dph; correlating to Stages 1, 2, 4 and juvenile, respectively; Sæle et al., unpublished) were killed by an anesthetic overdose of MS– 222 and fixed in 4% paraformaldehyde (PF) in 0.1 M Sørensen´s phosphate buffer (28 mM NaH_2_PO_4_, 72 mM Na_2_HPO_4_, pH 7.2) at 4°C for 30 to 48 hours depending on the larval size. Samples were rinsed in 1X phosphate buffered saline (PBS, pH 7.4) and treated overnight in 25% sucrose in 1X PBS/25% Tissue-Tek (O.C.T) Compound (Sakura Finetek Europe B.V, Netherland) at 4°C. Fixed larvae were then embedded in 100% O.C.T compound and maintained at– 80°C until sectioning. Transverse sections were cut at thickness of 10–12 μm using Leica CM 1850 cryostat, stretched on glass, dried at 65°C and stored at -80°C until used.

### *In situ* hybridization

Chromogenic *in situ* hybridization (CISH) was used to detect the location of NPY, CART, and pOX expression separately. Dual fluorescent *in situ* hybridization (FISH) was carried out on gene pairs (NPY/CART and pOX/CART) in the same sections. Hybridizations with NPY, CART and pOX digoxigenin or fluorescein labeled antisense riboprobes were revealed with either colorimetric NBT/BCIP staining (Roche Diagnostic), or fluorescent TSA and Fast Red staining (TSA PLUS Fluorescein Kit, PerkinElmer and Fast Red Roche Diagnostic, respectively). ISH was performed as described by Sandbakken M et. al [23], with some modifications as described below.

#### Pre-hybridization treatment

Transverse brain sections were air-dried, rehydrated and washed in 2X saline-sodium citrate (2X SSC), followed by proteinase K treatment (10 μg/mL in 100 mM Tris-HCl pH 8.0 and 50 mM ethylenediamine tetracetic acid (EDTA) pH 8.0) and post-fixation with 4% PF in 1X PBS (2 min each). Samples were then treated with 0.25% acetic anhydride in 0.1 M TEA (10 min) prior washing in 2X SSC (1 min), before incubation with riboprobes.

#### Hybridization

For CISH, 100 μl of hybridization solution (10% dextran sulphate, 10 mM Tris-HCl pH 7.5, 300 mM NaCl, 20 mM EDTA pH 8.0, 50% deionized formamide (Sigma-Aldrich), 0.2% tween-20, 1% blocking solution) with 200 ng digoxigenin or fluorescent labeled probes was applied to each slide and incubated overnight at 65°C in humid chamber with 2X SSC.

#### Post-hybridization

Following overnight incubation, sections were washed in 2X SSC (2x30 min) and treated with 50% deionized formamide in 2X SSC (65°C, 30 min), prior RNase treatment (RNase A 0.02 mg/ml in 10 mM Tris-HCl pH 7.5, 500 mM NaCl, 1 mM EDTA) (37°C, 30 min).

#### Color detection

For CISH, slides were washed with 2% blocking solution in 2X SSC- 0.05% triton x-100 (2 hours) and in 1X maleate buffer (100 mM maleic acid, 150 mM NaCl, pH 7.5; 2x5 min), before overnight incubation with 1:2000 anti-digoxigenin (FAB fragments) conjugated to alkaline phosphatase antibody (Roche Diagnostic) in antibody buffer (100mM maleic acid, 1% blocking solution, 0.3% triton x-100). Redundant antibody was removed by washes in 1X maleate buffer (2x10 min). Sections were washed in visualization buffer (100nM Tris-HCl pH 9.5, 5 mM MgCl_2_, 100 mM NaCl) for 10 minutes before being incubated with chromogen substrates Nitroblue Tetrazolium (NBT) and Bromo-4-chloro-3-indoylphosphate (BCIP; Roche Diagnostic) to develop the staining and then washed in stop buffer (10 mM Tris-HCl pH 7.5, 1 mM EDTA pH 8.0, 150 mM NaCl, pH 7.5) prior mounting in 60% glycerol in stop buffer, and stored at 4°C until visualization by Leica DM6000B microscope (4 and 20 dph) and Leica M420 microscope (46 and 76 dph) equipped with digital cameras Leica DFC 420 and Cool Snap-Pro color, respectively.

For dual florescence detection, sections were incubated overnight at 4°C with anti-fluorescein FAB fragments conjugated to horseradish peroxidase (POD) antibody (Roche Diagnostic) in antibody buffer (1:250). After washing in 1X maleate buffer, sections were rinsed in TNT washing buffer (100 mM Tris-HCl pH 7.5, 150 mM NaCl, 0.05% tween20) and incubated overnight at 4°C in darkness with fluorescein amplification reagent tyramide (TSA PLUS Fluorescein Kit, PerkinElmer) in TNT buffer (1:200). The reaction was then stopped in TNT buffer and the sections washed in immuno-buffer and 1X maleate buffer prior overnight incubation (4°C) with anti-digoxigenin (FAB fragments) conjugated to alkaline phosphatase antibody (Roche Diagnostic) in antibody buffer (1:2000). Fast red staining was performed by incubation with 0.05 mg naphtol substrate, 0.2 mg Fast Red chromogen, and 0.04 mg levamisole in 100 mM Tris-HCl pH 8.2 and 0.1% Tween-20 overnight at 4°C in darkness, according to manufacturer’s protocol (Fast Red tablets—Roche Diagnostic). Sections were then washed in 1X PBS, mounted with VECTASHIELD Mounting Medium (Vector laboratories) and analyzed with Leica TCS laser scanning confocal microscope using 10 and 20X oil immersion objectives. Image stacks of 1 μm optical sections were acquired with Leica PowerScan 2.5 software followed by brightness and contrast adjustments using Adobe Photoshop CS6 (San Jose, CA) before being mounted on panels.

### Ethics Statement

Austevoll Research station has the following permission for catch and maintenance of Atlantic cod: H-AV 77, H-AV 78 and H-AV 79, all issued by the Norwegian Directorate of Fisheries. Austevoll Research station has a general permission to run as a Research Animal facility and to conduct experiments involving all developmental stages of fish (code 93) provided by the national Animal Care and Use Committee (ACUC, the Norwegian Animal Research Authority, FDU, www.fdu.no). Although the sampling of stages of larval cod used in the current study do not require a specific permit according to Norwegian law of Animals in Research, Regulation of the 15^th^ of January 1996; the method of euthanization of fish larvae with the use of MS222 (metacaine) follows § 16 Euthanasia of Laboratory Animals stating that the selected euthanasia should involve no signs of unnecessary suffering of the animal.

This study is not categorized as a field study. The pond was merely used to filter different stages of mainly calanoid copepods which were used as feed for the fish larvae. The owner of the land has given permission to conduct such filtering. Calanoid copepods are not endangered or protected species and are not recognized as research animals in the Regulation on Animal Experimentation of 15. January 1996, last changed 6. August 2010, and no specific permission is therefore needed in order to use copepods as feed. The specific experiment described in this study was approved by the local representative of the Norwegian Research Animal Committee (Forsøksdyrutvalget, FDU, www.fdu.no) with the acceptance reference FOTS id 5448.

## Results

To increase our understanding of the gene distribution and putative roles of neuropeptides NPY, CART and pOX in selected brain regions of Atlantic cod larvae, we examined their mRNA expression patterns in serial brain sections by using both chromogenic and dual fluorescent *in situ* hybridization methods.

**Fig 1:**
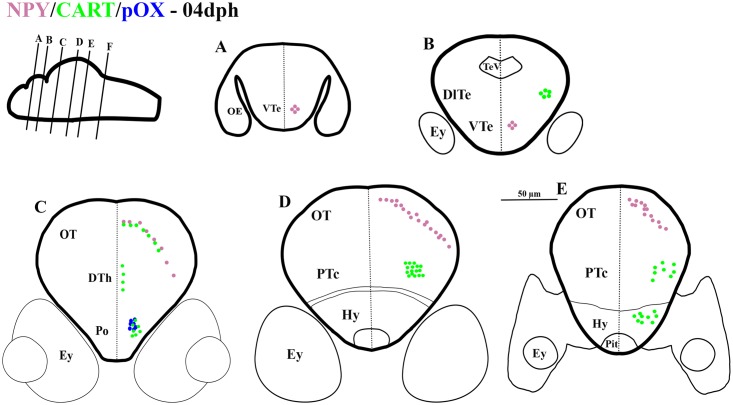
Schematic representation of rostro-caudal series of transverse sections of Atlantic cod brain at 4 days post hatch (dph); Stage 1. mRNA expression of NPY (purple dots), CART (green dots), pOX (blue dots), mRNA co-expression of NPY and CART (black dots), and pOX and CART (orange dots). Larval age (days post hatch; dph) correlates to Stage (based on Ø. Sæle, NIFES, Bergen, Norway, personal communication). Drawings in upper left corners show relative position of brain transverse sections.

**Fig 2:**
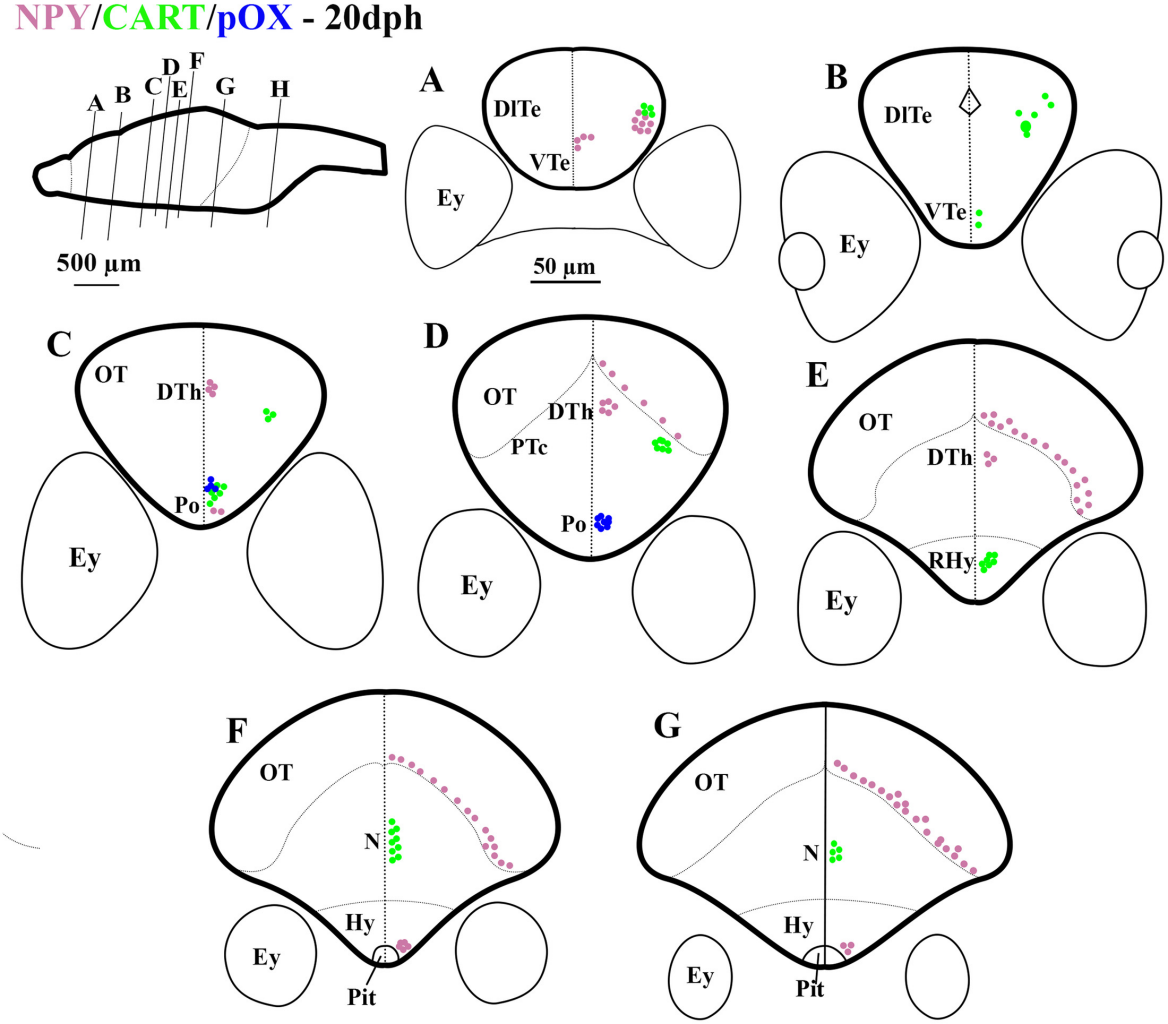
Schematic representation of rostro-caudal series of transverse sections of Atlantic cod brain at 20 days post hatch (dph); Stage 2. mRNA expression of NPY (purple dots), CART (green dots), pOX (blue dots), mRNA co-expression of NPY and CART (black dots), and pOX and CART (orange dots). Other captions as in Fig 1.

**Fig 3:**
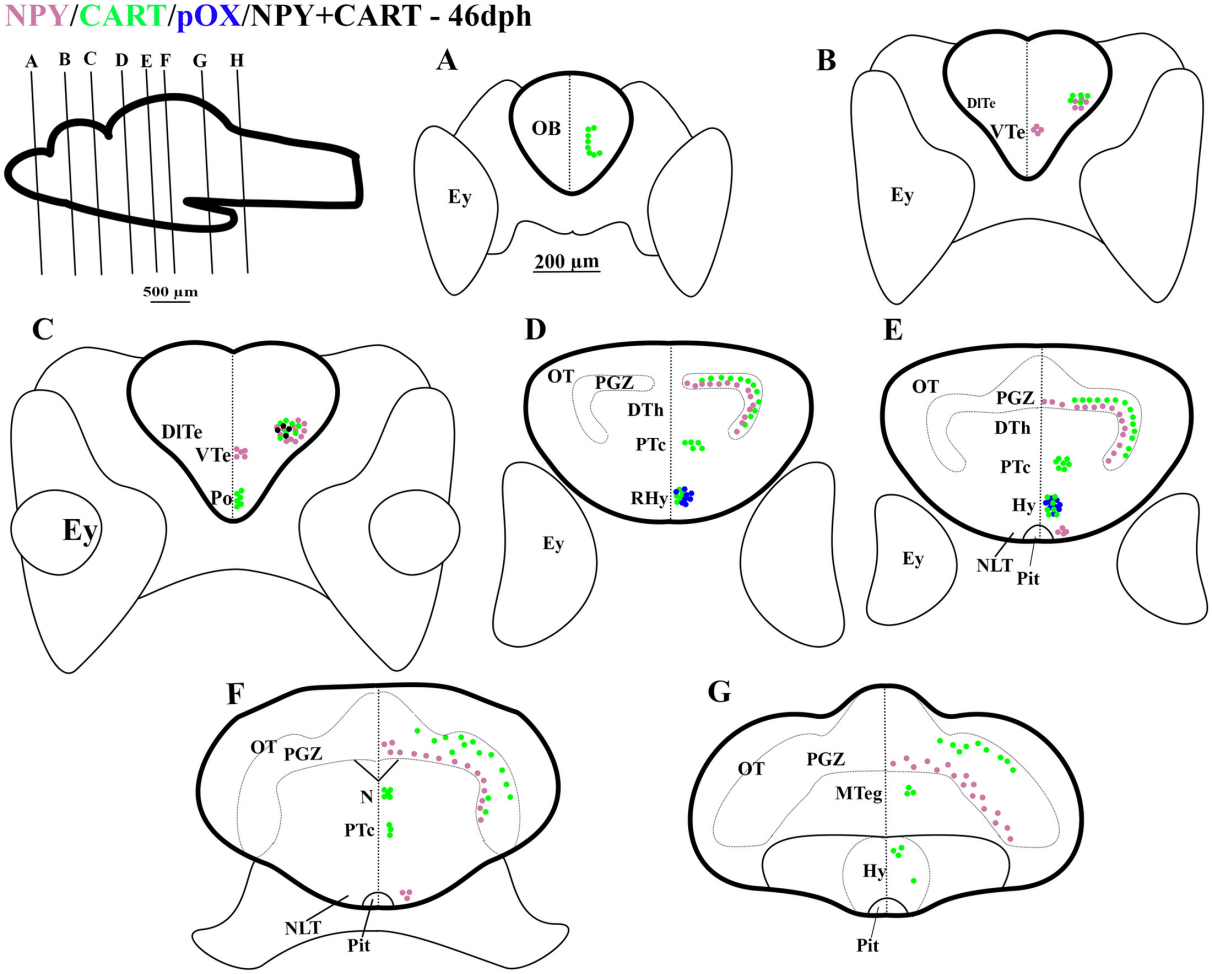
Schematic representation of rostro-caudal series of transverse sections of Atlantic cod brain at 46 days post hatch (dph); Stage 4. mRNA expression of NPY (purple dots), CART (green dots), pOX (blue dots), mRNA co-expression of NPY and CART (black dots), and pOX and CART (orange dots). Other captions as in Fig 1.

**Fig 4:**
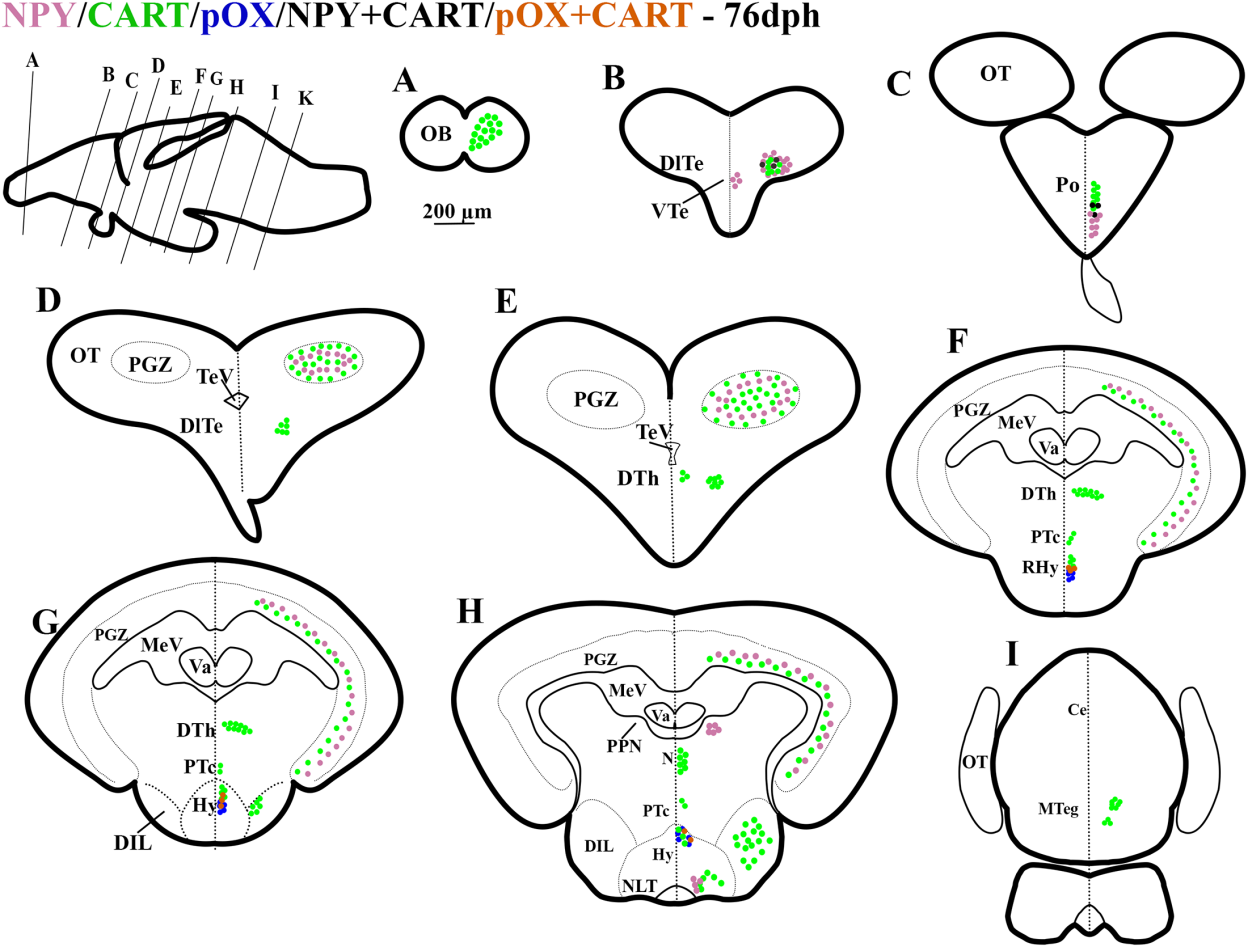
Schematic representation of rostro-caudal series of transverse sections of Atlantic cod brain at 76 days post hatch (dph); Stage Juvenile. mRNA expression of NPY (purple dots), CART (green dots), pOX (blue dots), mRNA co-expression of NPY and CART (black dots), and pOX and CART (orange dots). Other captions as in Fig 1.

Identification of brain regions and nuclei were mostly based on Atlas of Early Zebrafish Brain Development [24] and available literatures on similar studies in other teleost species [17, 25–29]. NPY, CART and pOX mRNA expression patterns in the forebrain and midbrain are summarized in Table 1 and Figs 1–4.

**Table 1:** Distribution of NPY, CART, and pOX mRNA brain expression by ISH methods in Atlantic cod at different larval developmental stages.

Brain region	Gene	04 dph	20 dph	46 dph	76 dph
**Telencephalon**	NPY	VTe	DlTe, VTe	DlTe, VTe	DlTe, VTe
	CART	DlTe	DlTe	OB, DlTe	OB, DlTe
	pOX	-	-	-	-
**Diencephalon & Mesencephalon**	NPY	Po, OT	Po, DTh, Hy, OT	Hy (NLT), OT (PGZ)	Po, Hy (NLT), PPN, OT (PGZ)
	CART	Hy, PTc	Po, RHy, PTc, N	Po, RHy, Hy, PTc, N, OT (PGZ), MTeg	Po, DTh, Hy (NLT, DIL), PTc, N, OT (PGZ), MTeg
	pOX	Po	Po	RHy, Hy	RHy, Hy

### NPY

NPY transcripts were detected in the telencephalon, diencephalon and optic tectum from 4 dph and its distribution pattern varied between all stages.

#### NPY mRNA in the telencephalon

**Fig 5:**
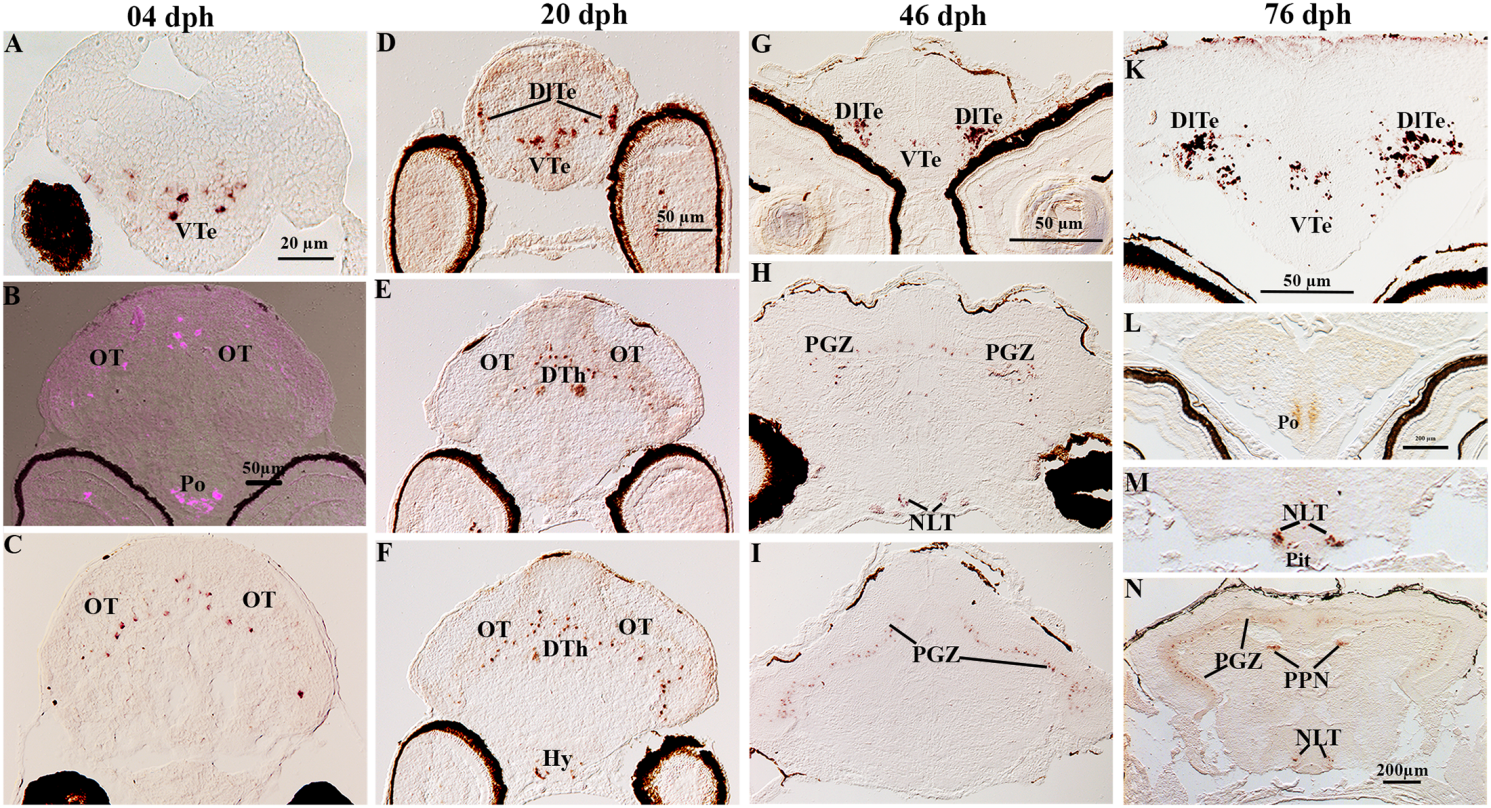
Localization of NPY gene expression in serial sections of brain in Atlantic cod larvae. At 4dph, (A) NPY mRNA expression in the ventral telencephalon (VTe), (B) several cells contained NPY gene expression in the ventral layer of the optic tectum (OT) and in the preoptic region (Po), (C) scattered cells expressing NPY mRNAs in the OT. At 20dph, (D) NPY mRNA expression in the lateral of dorsal telencephalon (DlTe) and in the VTe, (E) symmetric clusters of NPY expressing cells in the dorsal thalamus (DTh) and in the ventral layer of the OT, (F) hybridization signal in the ventral layer of the OT and in the hypothalamus (Hy). At 46dph, (G) symmetric clusters of NPY- expressing cells in the DlTe, (H) NPY in the periventricular gray zone (PGZ) and in the lateral tuberal nucleus of hypothalamus (NLT), (I) NPY in the PGZ of the caudal OT. At 76dph, (K) hybridization signal in the DlTe and VTe, (L) NPY- symmetric expression in the preoptic region (Po), (M) NPY symmetric expression in the NLT, (N) NPY symmetric expression in the PGZ of the OT, in the periventricular pretectal nucleus (PPN) and in the NLT.

At 4 dph larvae, most of the NPY mRNA expressing neurons were localized within the ventral telencephalon (VTe, Fig 5A), whereas at 20, 46 and 76 dph, two distinct symmetric clusters developed in the lateral part of the dorsal telencephalon (DlTe, Fig 5D, 5G and 5K). The telencephalon was the brain region showing the highest NPY mRNA- expression at all stages investigated.

#### NPY mRNA in the diencephalon and mesencephalon

In the preoptic region, NPY transcripts were found only in 4 and 76 dph old larvae (Po, Fig 5B and 5L). At 20, 46 and 76 dph, NPY mRNA containing neurons were also detected dorsally and laterally to the pituitary gland, in the presumptive lateral tuberal nucleus of the hypothalamus (Hy, Fig 5F; NLT, Fig 5H, 5M and 5N). NPY expression in the dorsal thalamus was only observed at the 20 dph stage (DTh, Fig 5E and 5F). At 76 dph, two symmetric clusters of NPY expressing cells were also observed within the presumptive periventricular pretectal nucleus (PPN, Fig 5N). The optic tectum exhibited NPY mRNA expression throughout its rostro-caudal brain extent, in its ventral layer at 4 and 20 dph (OT, Fig 5B, 5C, 5E and 5F) and in the periventricular gray zone at older stages (PGZ, Fig 5H, 5I and 5N).

### CART

CART mRNA showed a wide temporal distribution in the brain of the larvae, being expressed in both the telencephalon and diencephalon from 4 dph onwards. At 46 and 76 dph, new populations of CART positive neurons were also identified in the olfactory bulb, in the region of the nucleus of medial longitudinal fascicle (N), the periventricular gray zone of the optic tectum (PGZ) and in the midbrain tegmentum.

#### CART mRNA in the telencephalon

**Fig 6:**
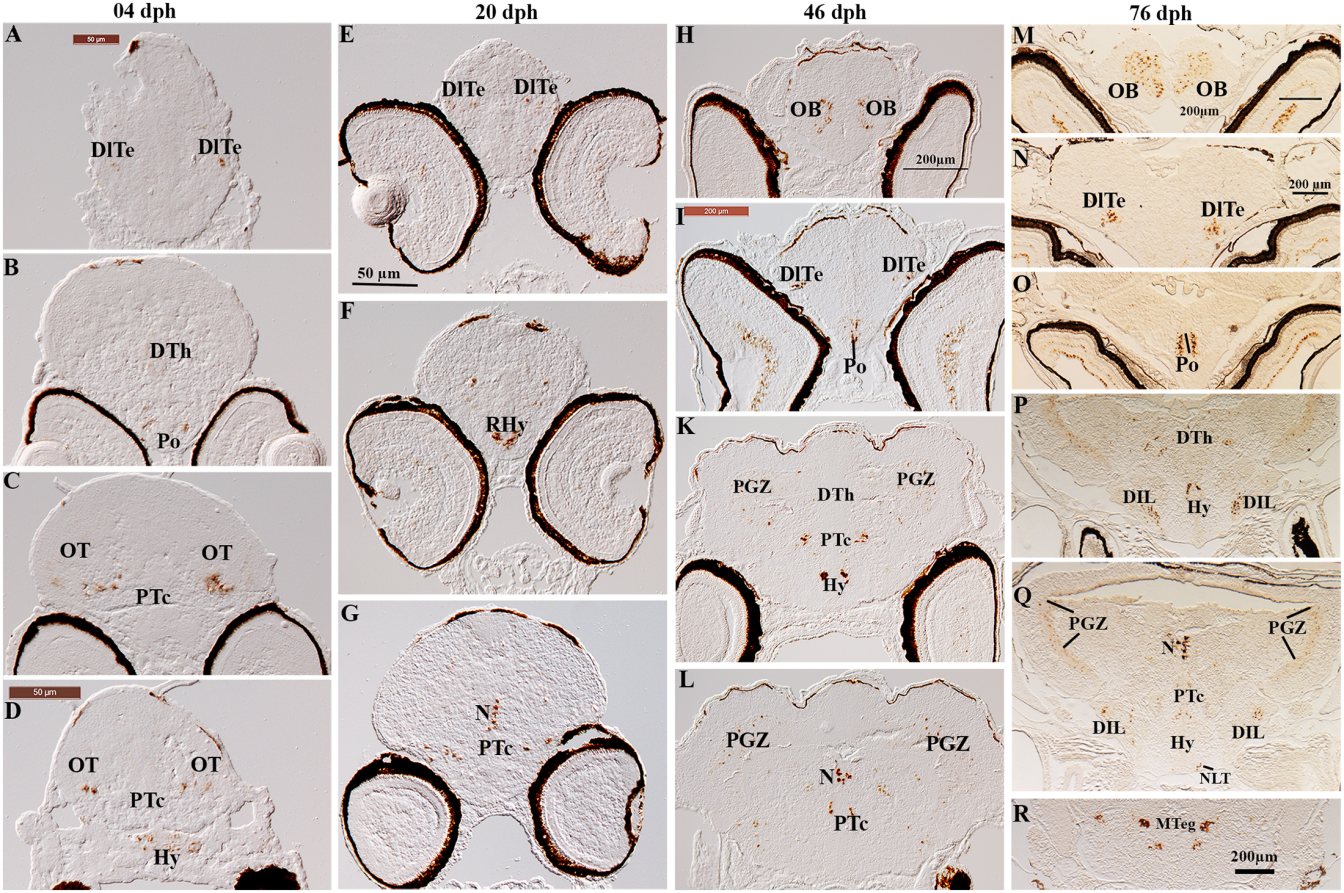
Localization of CART gene expression in serial sections of brain in Atlantic cod larvae. At 4dph, (A) CART positive cells in the lateral parts of dorsal telencephalon (DlTe), (B) CART mRNAs in the dorsal thalamus (DTh) and the preoptic region (Po), (C) symmetrical clusters of CART-containing neurons in the posterior tuberculum (PTc), (D) hybridization signal of CART in PTc and in the hypothalamus (Hy). At 20dph, (E) CART mRNAs in the DlTe, (F) CART expression in the rostral hypothalamus (RHy), (G) CART in the region of the nucleus of medulla longitudinal fascicle (N) and in the PTc. At 46dph, (H) two distinct clusters of CART mRNA- expressing cells in the olfactory bulbs (OB), (I) clusters of labeled neurons in the DlTe and in the Po, K: CART gene expression in the Hy, PTc and in the periventricular gray zone of the optic tectum (PGZ), (L) clusters of CART expressed cells in the region of the nucleus of medulla longitudinal fascicle (N). At 76dph, (M, N, O) show: CART mRNA- cells in the OB, in the DlTe and in the Po, respectively. (P) CART mRNA in the dorsal thalamus (DTh), Hy and in the diffuse nucleus of the inferior hypothalamic lobe (DIL). (Q) CART containing cells distribute in the PGZ, N and new clusters of labeled neurons in the putative lateral tuberal nucleus (NLT). (R) CART in the midbrain tegmentum (MTeg).

In the olfactory bulbs, two distinct populations of CART mRNA cells were visible at 46 dph larvae (OB, Fig 6H). The number of CART mRNA labeled cells greatly increased in two larger and rounded domains at 76 dph stage (OB, Fig 6M). Several scattered cells expressing CART were detected in the presumptive lateral part of the dorsal telencephalon (DlTe), at all stages investigated (Fig 6A, 6E, 6I and 6N).

#### CART mRNA in the diencephalon and mesencephalon

**Fig 7:**
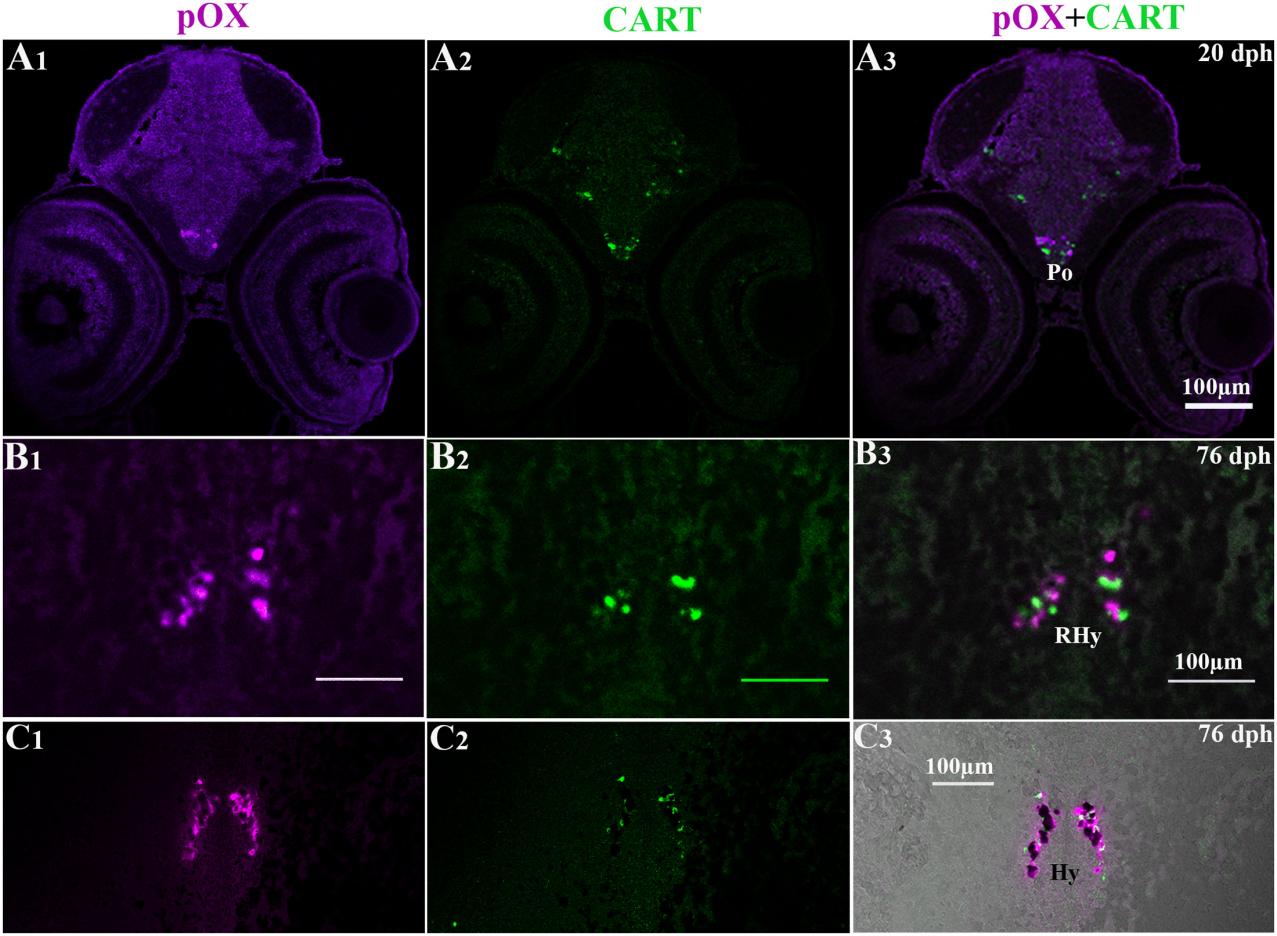
Dual FISH photomicrographs showing localization of pOX and CART gene expression in brain of Atlantic cod larvae at 20 and 76 dph. The left, middle, and right columns show fluorescent signals corresponding to red, green and overlap channels as reported above. (A_1-3_), pOX and CART mRNAs in the preoptic region (Po) at 20 dph; (B_1-3_), and (C_1-3_), in the rostral (RHy) and intermediate hypothalamus respectively, at 76 dph.

CART mRNAs had a wide distribution in the diencephalon, particularly in the preoptic region at all stages investigated (Po, Fig 6B, 6I and 6O for 4, 46, 76 dph, respectively and Fig 7A_3_ for 20 dph) and in the caudal and rostral hypothalamus at 4 and 20 dph, respectively (Hy, Fig 6D; RHy, Fig 6F). At 76 dph, CART- expressing neurons were found in the presumptive lateral tuberal nucleus (NLT, Fig 6Q) and in the diffuse nucleus of the inferior hypothalamic lobe (DIL, Fig 6P and 6Q). CART mRNAs- were also widely spread within the thalamus and in the posterior tuberculum. In the 4 dph larvae, a weak expression was seen in the dorsal thalamus (DTh, Fig 6B) while distinct symmetric clusters of CART positive neurons were detected in the presumptive lateral parts of the ventral posterior tuberculum (PTc, Fig 6C and 6D). From 20 dph onward, new populations of CART mRNA-cells were found in the presumptive nucleus of the medial longitudinal fascicle (N, Fig 6G, 6L and 6Q). At both 46 and 76 dph CART mRNAs were found in the presumptive periventricular gray zone of the optic tectum (PGZ, Fig 6K, 6L and 6Q) and in the midbrain tegmentum (MTeg, Fig 6R).

### pOX

**Fig 8:**
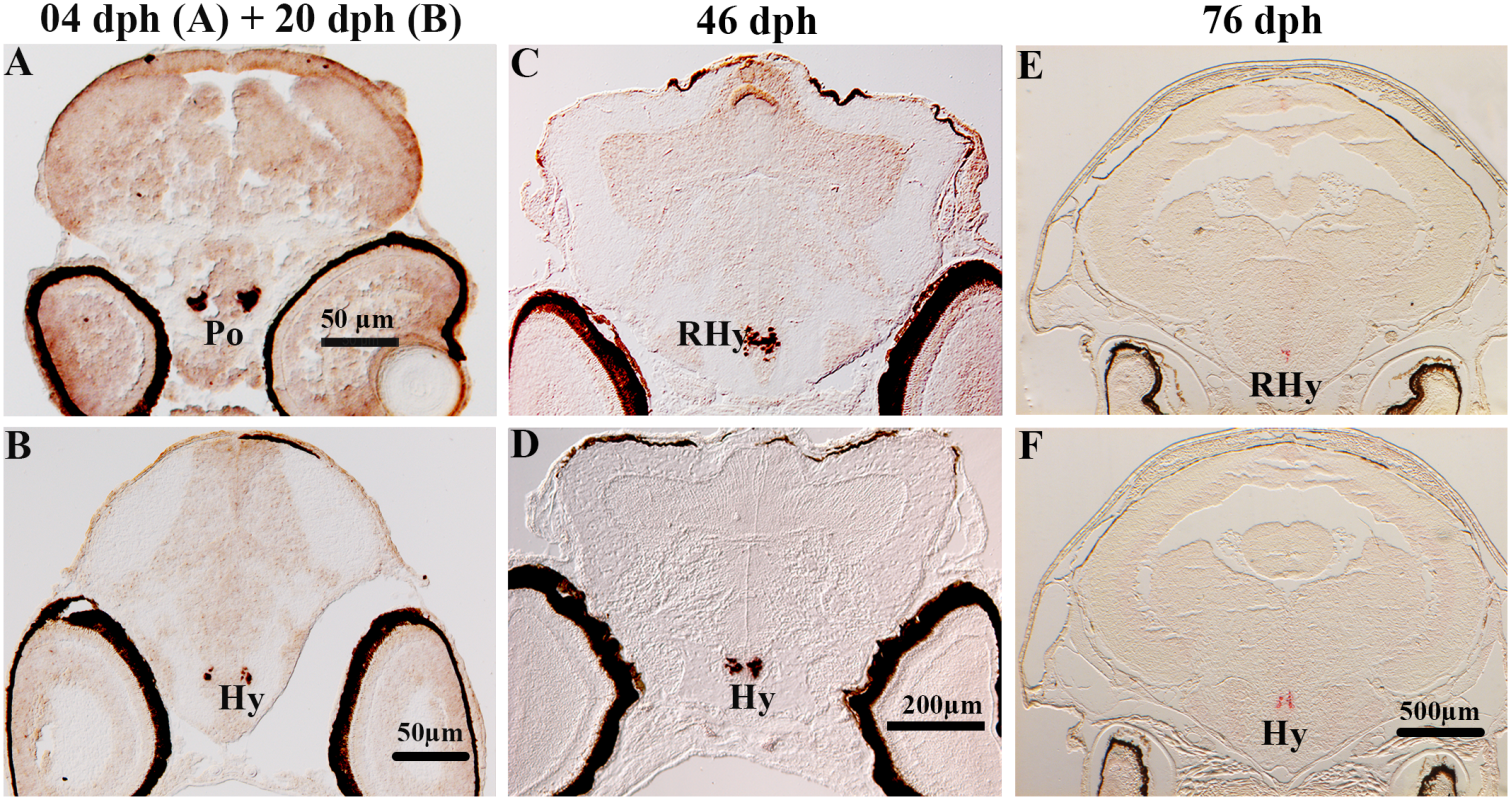
Localization of pOX gene expression in serial sections of brain in Atlantic cod larvae. ISH signals were detected in the preoptic region (Po) at 4 dph (A) and 20 dph (B) and in the rostral (RHy) and intermediate (Hy) hypothalamus at 46 dph (C, D), and 76 dph (E, F).

Prepro-orexin (pOX) had a restricted distribution in the cod brain throughout the four larval stages studied. At 4 and 20 dph, pOX mRNA were distributed in two clearly defined symmetric neuron clusters in the preoptic region (Po, Fig 8A and 8B). Later, at both 46 and 76 dph, these cell populations had a more spread distribution from rostral to intermediate regions of the hypothalamus (RHy and Hy, Fig 8C–8F).

### Co-localization of CART/pOX and CART/NPY in the brain of Atlantic cod larvae

**Fig 9:**
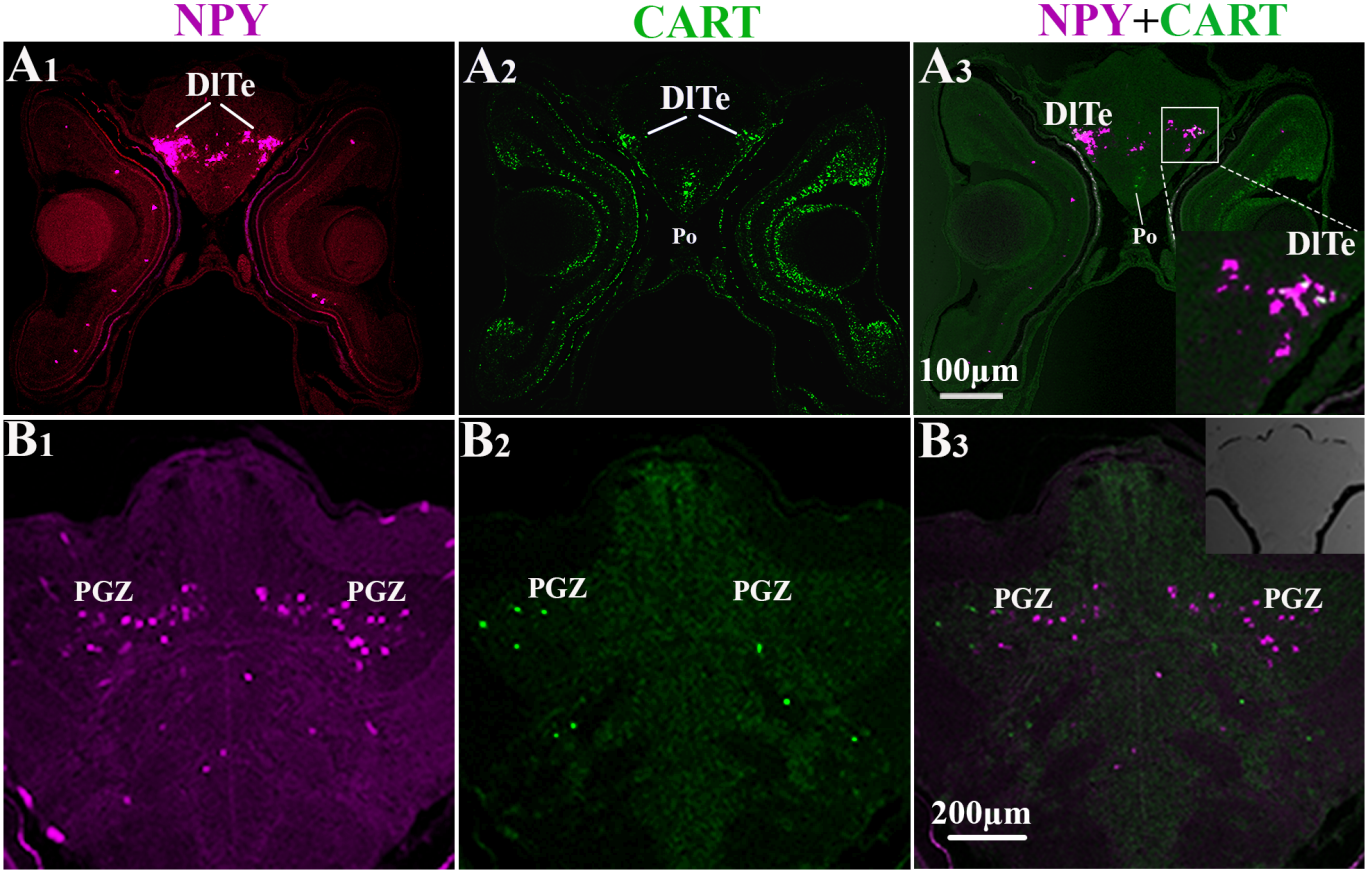
Dual FISH photomicrographs showing localization of NPY and CART gene expression in brain of Atlantic cod larvae at 46 dph. Fluorescence signals were recorded in the Fast Red (NPY, left column; detection of wavelengths greater than 560 nm) and TSA (CART, middle column; detection of wavelengths from 500 nm to 540 nm) detection channels. Right column shows both channels overlap to visualize co-localization of NPY and CART gene expression (yellow). (A_1-3_), NPY and CART mRNAs in the lateral parts of the dorsal telencephalon (DlTe); (B_1-3_), in the periventricular gray zone of the optic tectum (PGZ).

On the basis of single-gene detection studies presented here, dual fluorescent ISH protocol was additionally used to detect potential mRNA co-localization of CART/pOX and CART/NPY in same brain sections and at the level of single neurons. Dual fluorescent ISH of CART/ pOX genes identified adjacent CART and pOX expressing cells in the preoptic region at 20 dph (Po, Fig 7A_1-3_). Interestingly, at 76 dph, CART and pOX mRNA-expressing cells shared cellular domains in the intermediate hypothalamus where some cells clearly co-expressed both genes (Fig 7B_1-3_ and 7C_1-3_). At 46 dph, both CART and NPY expressing cells were detected in the lateral parts of the dorsal telencephalon (DlTe, Fig 9A_1-3_) and in the periventricular gray zone of the optic tectum (PGZ, Fig 9B_1-3_). Particularly, mRNA co-localization was seen in few neurons of the DlTe (Fig 9A_3_).

## Discussion

The present study describes for the first time the development expression patterns of NPY, CART and pOX genes in discrete brain regions of the marine teleost Atlantic cod, from start of exogenous feeding until juvenile stage. The mRNA distribution indicates that all three brain regions investigated (telencephalon, diencephalon and mesencephalon) contain both orexigenic and anorexigenic key elements in appetite control. The spatial and temporal expression patterns indicate a progressive development of the brain regulatory networks that control appetite during larval ontogeny.

### NPY

The NPY mRNA expression changed spatially during larval development. At the first feeding stage (4 dph), high NPY mRNA expression levels were found in the ventral telencephalon, diencephalon, and optic tectum. From Stage 2 (20 dph) to the juvenile stage (76 dph), two distinct and growing symmetric clusters of labeled cells appeared in the lateral part of ventral telencephalon. From 20 dph onwards NPY- gene expressing cells were also seen in the ventrolateral parts of hypothalamus. In the mesencephalon, NPY mRNA was found throughout the rostro-caudal extent of the optic tectum in the presumptive periventricular gray zone at 4 and 20 dph and in the same developed region at older stages.

NPY RT-PCR expression during early developmental stages of fish has also been previously reported; for instance at 3 dph in Atlantic cod larvae [12], at blastula stage in orange spotted grouper [19] and at zygote stage in blunt snout bream (*Megalobrama amblycephala*) [30, 31]. Moreover, immunoreactivity-studies in zebrafish have shown that NPY-ir cell bodies appeared for the first time at 60 h post fertilization (hpf) in the hypothalamus [26]. NPY-ir neurons were also found in the olfactory placode at 16 dpf in chum salmon (*Oncorhynchus keta*) [31], and in 40 mm-staged embryos of cloudy dogfish (*Scyliorhinus torazame*) [31]. Thus, it is likely that ontogeny of the NPY system, both at mRNA and protein levels, is required for a proper brain development at very early embryonic stages.

The telencephalon showed the highest NPY expression at all stages investigated, in accordance with previous studies in juvenile Atlantic cod [32] and other fish species at adult stages (e.g. sea bass *Dicentrarchus labrax* [17], goldfish (*Carassius auratus*) [18, 28, 33, 34], winter skate (*Raja ocellata*) [35], killifish (*Fundulus heteroclitus*) [36], African lungfish (*Protopterus annectens*) [37, 38], and white sturgeon (*Acipenser transmontanus*) [39, 40]. Due to the proposed role of NPY as an appetite stimulator in fish [11, 41, 42], this could indicate that the telencephalon may possess some components in hunger regulation in Atlantic cod and that this system is already present from onset of exogenous feeding.

The expression of NPY mRNAs in the diencephalon is in agreement with previous studies that report NPY mRNA in the preoptic region from the early larval stages, juvenile and adult fish (e.g. goldfish [18, 34], sea bass [17], killifish [36], African lungfish [37], and gilthead seabream (*Sparus aurata*) [43]). ISH signals were mainly observed in the ventro-lateral parts of hypothalamus, referred to as the putative NLT at the two older stages investigated. In previous studies, NPY-ir and/or mRNA transcript detection in NLT were observed in sea bass [17], dogfish (*Scyliorhinus canicula*) [44], white sturgeon [39], killifish [36], and pejerrey (*Odontesthes bonariensis*) [45].

In goldfish, NPY mRNA is widely distributed in the thalamus [18]. In contrast, we found NPY expression only in the dorsal thalamus at 20 dph stage. Given the organization of the pretectum in cichlid fish [27, 46], the two clusters of NPY mRNA expressing cells identified in the dorsal midbrain of cod larvae (Fig 5N) may correspond to the periventricular pretectal nucleus (PPN), particularly to the ventral periventricular pretectal nucleus (PPv). Similar observations have also been reported in adult sea bass [17].

In agreement with our results in Atlantic cod larvae, NPY mRNA expression has been observed within the optic tectum of goldfish [18, 33], sea bass [17] and winter skate [47], and numerous studies have reported that NPY- ir cells and/or fibers appear in the optic tectum in goldfish [28, 34], in Atlantic salmon [48, 49], in pejerrey [45], in gilthead seabream [43] and in lamprey *Petromyzon marinus* [50].

### CART

In the present study, CART mRNA expression was detected in the telencephalon, preoptic region and diencephalon from 4 dph onwards. After metamorphosis, at 46 and 76 dph, CART was also found in olfactory bulb and optic tectum. The early mRNA expression found in the telencephalon and its late appearance in the olfactory bulbs is in agreement with a previous study on zebrafish where CART immunoreactivity (ir) cells in the telencephalon were detected as early as 24 hpf, while in the olfactory bulbs they first appeared at 46 hpf [25]. We found the highest level of CART mRNA in the olfactory bulbs at the latest larval stage investigated, also in agreement with previous observations in goldfish [21]. Taken together, these results suggest that the CART peptide may be associated with the regulation of feeding in the early larval stages and might have additional roles in sensory (olfactory and visual) information/transmission at later stages.

The expression of CART mRNA in the diencephalon showed interesting spatio-temporal patterns during cod larval development. CART mRNAs were initially found in the preoptic region as early as 20 dph, with expression gradually increasing at later stages [25]. The preoptic region is known to play an important role in both reproduction and feeding, in fish [51, 52] as in mammals [53]. In the sexually inmature cod larvae the early onset of CART mRNA expression in the preoptic region may therefore suggest that this region aquire an involvement in feeding behavior from the early larval stages.

CART mRNA-expressing cells were detected in rostral and intermediate hypothalamus from 4 dph onwards. The hypothalamic domains expressing CART mRNA at 76 dph were identified the presumptive NLT and the DIL. Similarly, in zebrafish CART(ir) fibers in the hypothalamus were first detected at 4 dpf in rostral, intermedial, and central zones of the periventricular hypothalamus, DIL, and lateral hypothalamus [25].

The high expression of CART, combined with the absence of the orexigenic modulator NPY in the DIL, suggest that this nucleus may serves as a “satiety center” in Atlantic cod. DIL may be the functional homologue to the mammalian ventromedial nucleus (VMN) situated in the intermediate hypothalamus and associated with satiety control [54, 55].

We also observed a wide distribution of CART mRNAs in the thalamus and in the posterior tuberculum (PTc) especially from 46 dph, and new groups of labeled cells appeared in the putative (N) from 46 dph onwards. The presence of CART mRNA expression in the thalamus of cod larvae is in agreement with previous studies on CART-(ir) cell distribution in other teleost fish [25, 56]. In fish, thalamic nuclei are involved in relaying sensory inputs to the telencephalon [57], and the wide distribution of CART mRNAs we found in the same brain regions reinforces the idea that this peptide may be related to sensory information processing in cod larvae as well.

CART mRNA-expressing cells were also detected in the optic tectum and in the midbrain tegmentum, from 46 dph onwards. The teleostean optic tectum is the primary target of the optic nerve fibers [57]. Its involvement in processing of visual information has been shown well documented in teleost species [29, 58]. The detection of CART mRNAs in the ganglion cell layer (GCL) in the present study (data not shown) could indicate that the CART peptide is involved in visual processing.

In summary, CART mRNA expression was apparent at the start of feeding and the occurrence of a wide and dense CART expression in thalamus and hypothalamus suggests that neuronal cell populations identifying key components controlling satiety may be located in these areas. Moreover, the presence of CART mRNA in the olfactory bulb and optic tectum suggests that CART functions outside that of appetite control, perhaps modulating sensory systems.

### pOX

The pOX mRNA expression was exclusively located in the preoptic region at 4 and 20 dph, and extended to the hypothalamus from 46 dph onwards. The hypothalamic pOX expression in our study is consistent with other ISH studies in zebrafish at both embryonic to larval stages [59] and in adults [20, 60, 61]. However using RT-PCR methods in adult Atlantic cod, pOX mRNAs were detected in several other brain regions including the olfactory bulb, telencephalon, hypothalamus and optic tectum [16]. Similar RT-PCR expression profiles were also found in mature specimens of winter skate [62], winter flounder [63], orange-spotted grouper [15] and cichlids [20]. In adult fish, the pOX peptide has been demonstrated using immunohistochemistry in the ventral telencephalon, thalamus, and hypothalamus of goldfish [64], extensively throughout the brain in medaka [61], and in the preoptic region and hypothalamus in cichlids [20]. Together, these results indicate that in adult fish pOX transcripts are detected in several brain regions while at early embryonic and larval stages pOX expression/functions are primarily exerted through neurons located in the preoptic area and hypothalamus. As environmental challenges and related feeding behaviors become more complex in adult fish, it is reasonable that the mechanisms underlying these brain functions may require more sophisticated neurochemical and anatomical brain networks.

### Co-localization of CART/NPY and CART/pOX in the brain of Atlantic cod larvae

CISH for single-gene detection indicated some potential sites of co-localization of mRNAs for CART and NPY or pOX genes. DFISH detection protocol was implemented using combination pairs of the anorexigenic factor CART with either NPY or pOX orexigenic genes in the same brain sections. The distribution of both NPY and CART mRNAs by DFISH in the telencephalon and periventricular gray zone of the optic tectum were consistent with results recorded by CISH. Numerous NPY and CART mRNA positive neurons were detected in same brain regions, e.g. telencephalon and optic tectum, however only few neurons co-expressing these two genes were found in the DlTe.

Both NPY and CART mRNA transcripts were found in the lateral tuberal nucleus (NLT) at 76 dph juveniles. In mammals, the hypothalamic nucleus that releases both orexigenic and anorexigenic peptides is the arcuate nucleus [65], suggesting that the NLT might be the teleostean homolog of the mammalian arcuate nucleus (ARC) [66, 67].

Interestingly, we also found clear co-localization of CART and pOX mRNAs in hypothalamic neurons of the caudal part of pOX cell population, which overlaps with the rostral part of CART cell population. The co-expression of CART and NPY in the same neurons supports the idea that this hypothalamic area exerts a multifunctional control of appetite.

Overall, CISH and DFISH data show a wide distribution of NPY, pOX, and CART expression in the hypothalamus of cod larvae substantiating its central role in control of food intake as previously showed in other teleost fish [17,45] and mammals [9–11].

Moreover, the temporal and spatial differences in NPY, CART, and pOX mRNA expression from first feeding stage (4 dph), through metamorphosis and until juvenile (76 dph) stages indicate a dynamic distribution consistent with progression of brain development and function. These observations support the statement that the neurotransmitter systems underlying appetite control continues to develop during the larval stages of teleosts as well as after the onset of exogenous feeding [68, 69].

## Abbreviations

CART: cocaine- and amphetamine-regulated transcript
Ce: cerebellum
DIL: diffuse nucleus of the inferior hypothalamic lobe
DlTe: lateral part of the dorsal telencephalon
DMN: dorsomedial nucleus of hypothalamus
DTh: dorsal thalamus
Ey: eye
Hy: hypothalamus
MeV: mesencephalic ventricle
MTeg: midbrain tegmentum
N: region of the nucleus of medial longitudinal fascicle
NLT: lateral tuberal nucleus of hypothalamus (in teleost)
NPY: neuropeptide Y
OB: olfactory bulb
OE: olfactory epithelium
OT: optic tectum
PGZ: periventricular gray zone of the optic tectum
Pit: pituitary
Po: preoptic region
pOX: prepro-orexin
PPN: periventricular pretectal nucleus
PTc: posterior tuberculum
PVN: paraventricular nucleus
RHy: rostral hypothalamus
TeV: telencephalic ventricle
Va: valvula cerebelli
VMN: ventromedial nucleus of the hypothalamus
VTe: ventral telencephalon
